# Application of contrast-enhanced ultrasonography in the diagnosis of post-kidney transplant lymphoproliferative disorder in native kidney- a case report

**DOI:** 10.1186/s12885-019-6355-0

**Published:** 2019-11-21

**Authors:** Jian-Chao Zhang, Hui-Xia Lan, Hui-Juan Zhao, Yang-Yang Lei, Li Ma, Xiao-Yan Xie, Ming-de Lu, Wei Wang

**Affiliations:** 1 Department of Medical Ultrasonics, Institute of Diagnostic and Interventional Ultrasound, The First Affiliated Hospital of Sun Yat-Sen University, No.58 Zhongshan Road 2, Guangzhou, 510080 People’s Republic of China; 2 Department of Medical Ultrasonics, Guangdong Second People’s Hospital, Haizhu District, Guangzhou, 510317 People’s Republic of China

**Keywords:** Contrast-enhanced ultrasonography, Biopsy, Post transplantation lymphoproliferative, Kidney transplantation

## Abstract

**Background:**

Post-transplant lymphoproliferative disorders (PTLDs) represent a spectrum of heterogenetic lymphoid proliferations. PTLD is a serious complication that affects the long-term survival of kidney transplant patients. Imaging examination is an important method for detecting and diagnosing PTLD. Contrast-enhanced ultrasonography (CEUS) and CEUS-guided biopsy are important modalities for tumor detection and diagnosis. In this case, we describe a 69 years old man in whom a native kidney PTLD was confirmed by CEUS.

**Case presentation:**

A 69-year-old male patient who had a kidney transplant 1 year earlier presented with 3 months of progressive myasthenia of both lower limbs associated with amyotrophy and weight loss. Although positron emission tomography/computed tomography (PET-CT) showed a high metabolic lesion in the untransplanted kidney, abdominal contrast enhanced computed tomography cannot detect the lesion in the atrophic left kidney. The above examinations showed that the transplanted kidney was normal. CEUS can detect a homogeneously enhanced lesion in the same location as PET-CT. Subsequently, a biopsy was performed under CEUS guidance, and the final pathological diagnosis was diffuse large B-cell lymphoma. The patient then received the R-CHOP treatment. Unfortunately, pulmonary thromboembolism occurred 2 weeks later, and the patient’s condition was not alleviated through active treatment. Finally, the patient’s family gave up treatment, and the patient was discharged.

**Conclusion:**

The case suggested that CEUS was a valuable imaging method for patient with renal transplantation to detect and diagnose of PTLD.

## Background

Post-transplant lymphoproliferative disorders (PTLDs) represent a group of heterogenetic lymphoid proliferations ranging from polyclonal lymphoid proliferation to lymphomas. PTLD occurs after solid organ transplantation and is associated with administration of immunosuppressive agents [1]. Prompt diagnosis of PTLD is critical to prognosis, to prevent the further development of malignant lymphoma [2]. Imaging examination is an important method to detect and diagnose PTLD, including conventional ultrasonography (US), Doppler ultrasound, computed tomography (CT) and magnetic resonance imaging (MRI). Contrast-enhanced computed tomography (CE-CT) and contrast-enhanced magnetic resonance imaging (CE-MRI) are important image modalities for characterizing PTLD [3, 4]. However both the iodinated contrast agent for CE-CT and the gadolinium for MRI have potentially nephrotoxic, restricting their use in patients with impaired renal function [5]. Compared with CE-MRI and CE-CT, the contrast agents used for contrast-enhanced ultrasonography (CEUS) are no nephrotoxic and can be safety applied to patients with renal dysfunction. CEUS can also provide real-time visualization of contrast-enhanced patterns, which can be used for differential diagnosis of renal lesions [6]. In addition, the exact real-time aspect of CEUS makes it uniquely suited for interventions [7]. CEUS therefore has gradually become the preferred method to detect and diagnose renal tumors in chronic kidney disease and post-transplant patients in recent years [8, 9]. We report a case of a 69 year old man with a native renal lymphoma associated with PTLD. We describe the contrast-enhanced features of the tumor and the application of a CEUS guided biopsy on the tumor, which is not well visualized using US. To the best of our knowledge this is the first report on CEUS manifestations of native renal lymphoma following renal transplantation.

## Case presentation

**Fig. 1:**
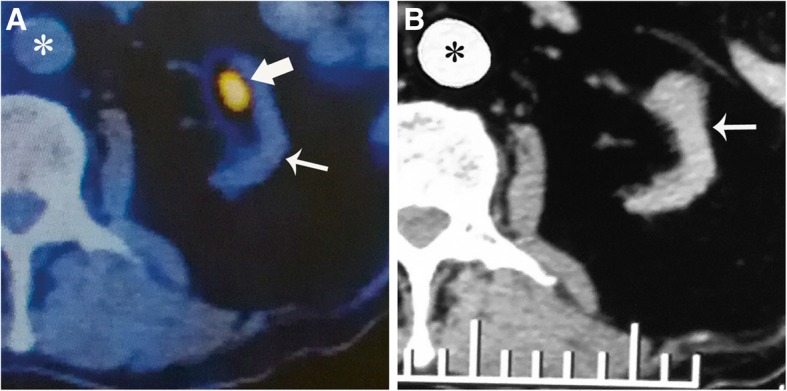
Positron emission tomography/computed tomography (PET-CT) and computed tomography (CT) images of the outer hospital. **a** PET-CT revealed a 1.4 cm intense FDG uptake (Standardize Uptake Values max: 4.7) lesion (wide arrow) at the medial portion of the left native kidney; **b** AS the same level of PET-CT, contrast-enhanced CT did not detect any solid lesion in the left native kidney (narrow arrow)

A 69-year-old male patient with kidney transplantation was submitted to our hospital for further evaluation and treatment of a left native kidney mass. The patient had chronic kidney disease for 2 years and had undergone dialysis for 10 months before kidney transplantation. The patient had a 20 years history of hypertension (the highest blood pressure: 170/106 mmHg) without diabetes. The patient underwent kidney transplantation 11 months prior to our study, and maintained a triple immunosuppressive regimen that consisted of tacrolimus, mycophenolatemofetil and prednisolone after transplantation. Two months prior, the patient was submitted to the local hospital for progressive myasthenia both lower limbs and weight loss. Routine laboratory tests showed the following: creatinine and urea were normal, but Epstein-Barr virus (EBV) and cytomegalovirus (CMV) IgG antibodies were positive. An MRI of the lumbar showed multiple vertebral bone destruction. A positron emission tomography/computed tomography (PET-CT) scan was then performed for further information, showing an intense FDG accumulation lesion (approximately 1.4 cm, standardized uptake values max: 4.7) in the medial portion of the left untransplanted kidney (Fig. 1a). PET-CT then proposed possible diagnosis of a renal malignant tumor and bone metastasis, but it could not confirm diagnosis. CE-CT was performed with following scanning parameters: tube voltage, 120 kV; tube current, 250 mA; and slice thicknesses, 1 mm and 10 mm. CE-CT (Fig. 1b) and US detect multiple cysts only and cannot find a solid lesion proposed by PET-CT. PET-CT and CE-CT did not reveal any abnormalities in transplanted kidney. To further evaluate the tumor and definite diagnosis, the patient was sent to our department.

**Fig. 2:**
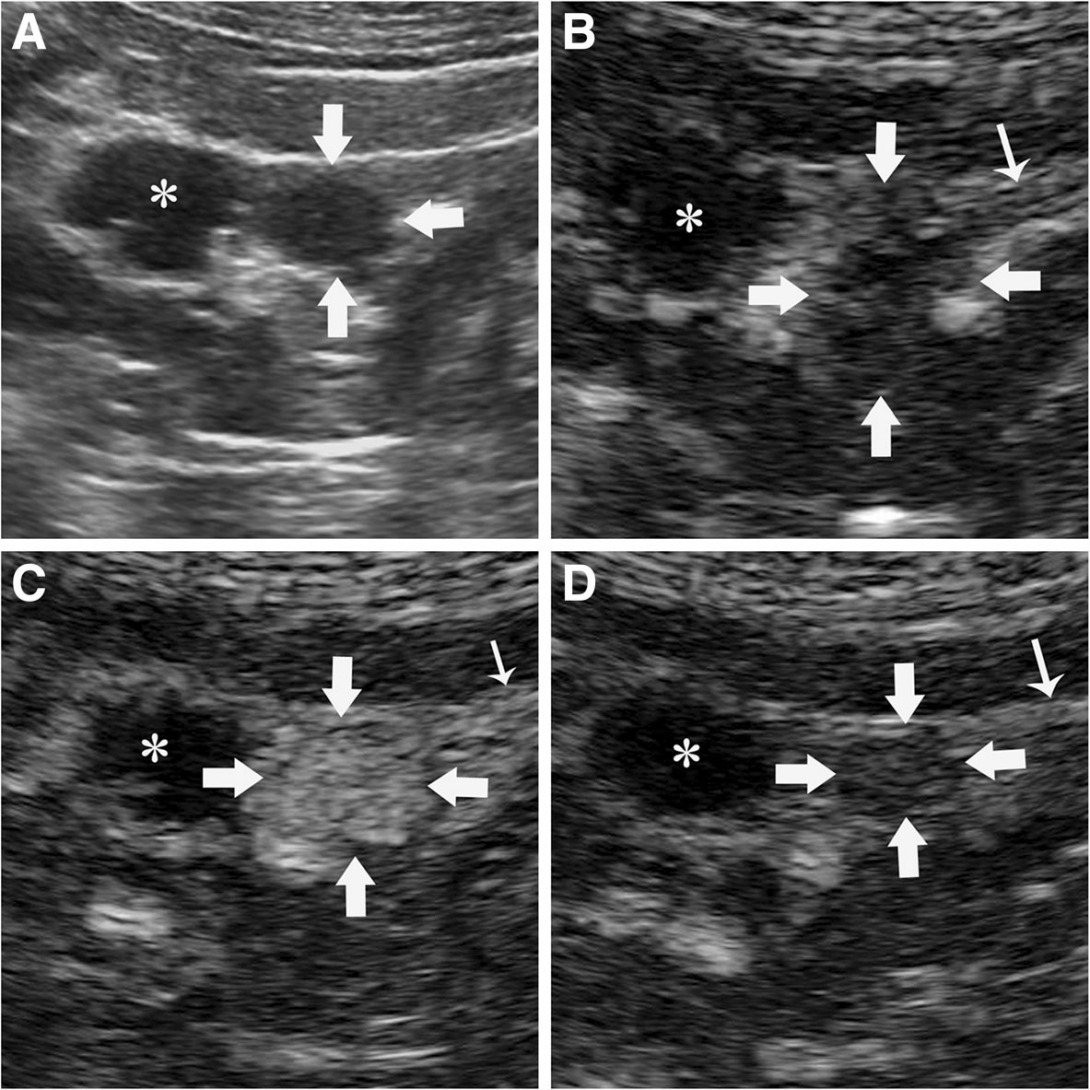
The contrast-enhanced ultrasonography features of the tumor in native left kidney .**a** the tumor (arrow) and cysts (asterisk) are presenting hypoechoic lesions in conventional ultrasonography which is difficult to differentiate from surrounding cysts. **b**. After injection of contrast agent, the edge of tumor (wide arrow) start enhancing at 20 s, showing hypo-enhancement as surrounding parenchyma. **c** The lesion achieved peak-enhancement at 33 s, more intense than surrounding kidney parenchyma (narrow arrow). **d** The lesion gradually turned to hypo-enhancement at 90 s, showing hypo-enhancement in comparison to surrounding parenchyma .The cyst (asterisk) around the lesion still non-enhancing during whole examination

**Fig. 3:**
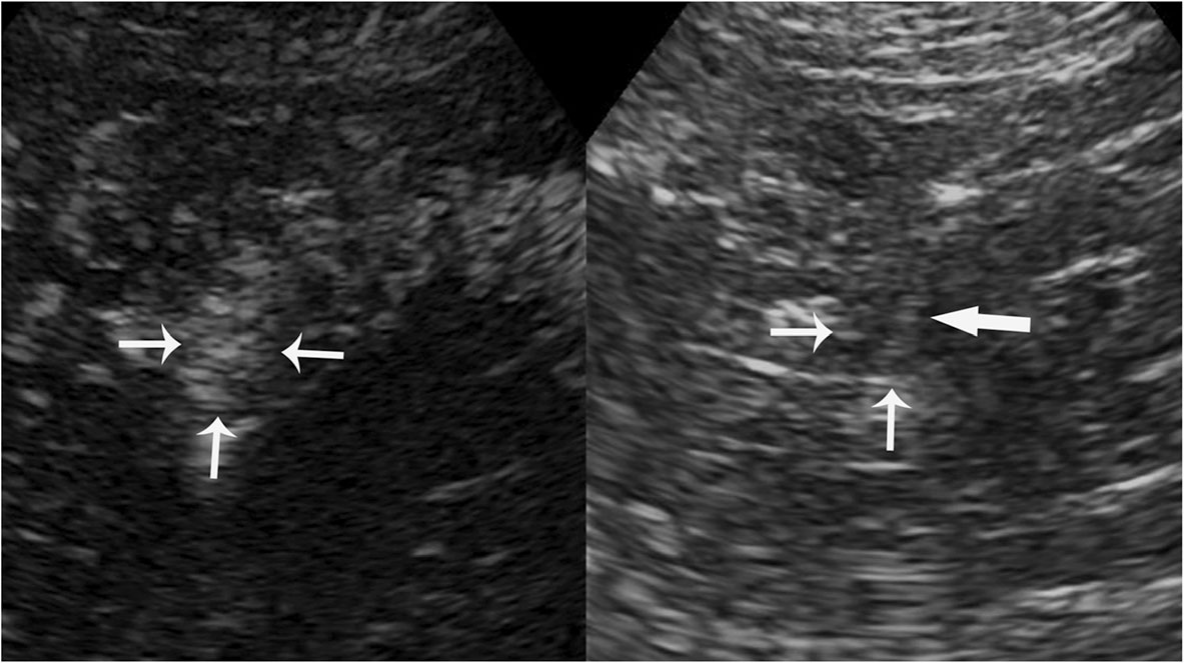
Contrast-enhanced ultrasonography guided biopsy of the mass. The needle tip (wide arrow) has correctly been inserted into the completely enhanced mass (narrow arrow)

**Fig. 4:**
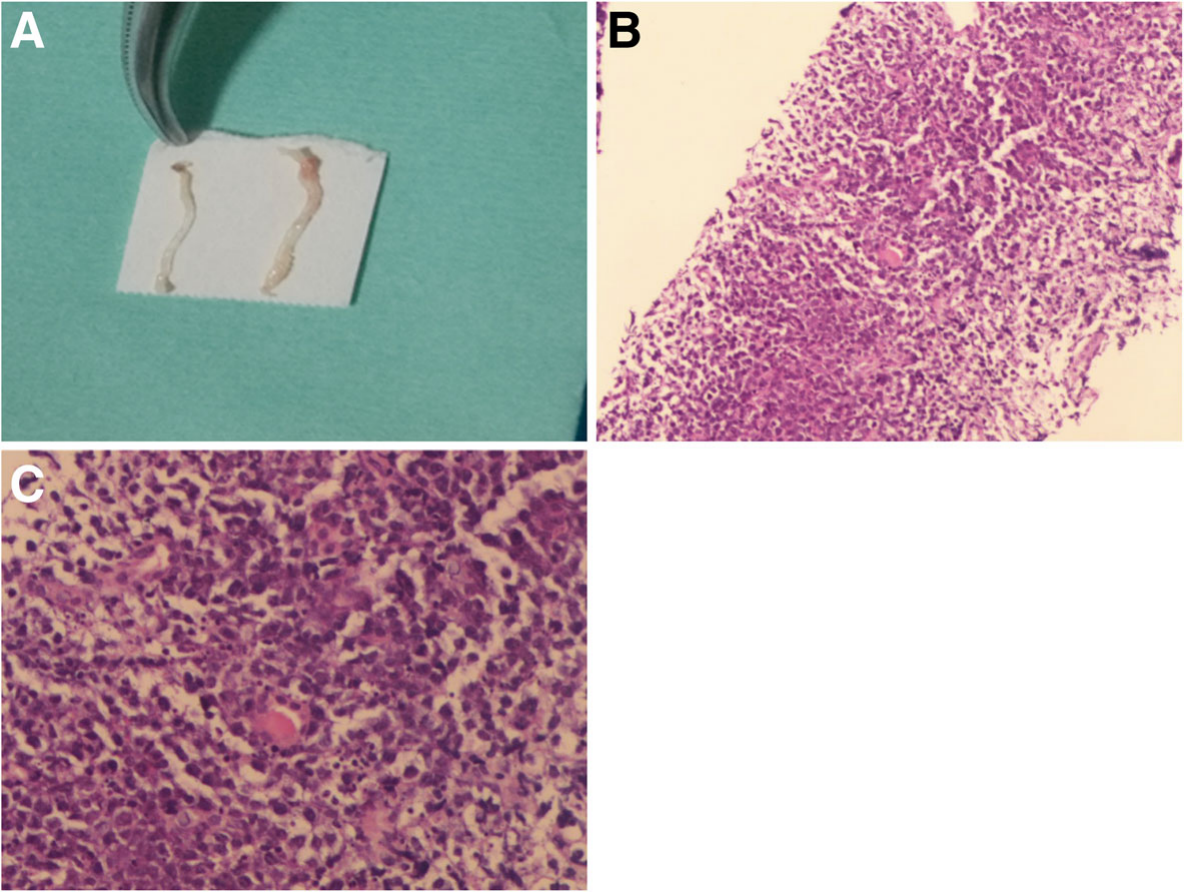
Histological findings of the core needle biopsy specimen from the mass. **a** The core needle biopsy specimen from the mass; **b**, **c** Hematoxylin and eosin staining of specimen from the mass revealed diffuse infiltration of atypical lymphoid cells. (B 10×,C 40×)

Ultrasonography was performed using the Aplio500 (Toshiba Medical Systems, Tokyo, Japan) equipped with a 375BT convex transducer (frequency range 3.0–6.0 MHz). The transplanted kidney was located in the right iliac fossa, and showed no abnormalities in conventional B-mode and Doppler ultrasonography. Only atrophic untransplanted kidneys with multiple cystic lesions were observed in US (Fig. 2a). Then a bolus of 2.4 ml of SonoVue (Bracco, Milan, Italy) was administered intravenously and flushed by 5.0 ml of 0.9% saline was performed. The examination was performed at a low mechanical index of 0.09. CEUS revealed a completely homogeneously enhancement lesion (approximately 1.3 × 1.1 cm) in the medial portion of the left untransplanted kidney in 33 s post-injected of contrast agent. Enhancement had progressed from the periphery towards the center of the lesion at 20 s post-injection (Fig. 2b). The lesion exhibited completely enhancement at 33 s (Fig. 2c) and was slightly higher enhanced than the surrounding parenchymal. The lesion gradually turned to hypo-enhancement at 90 s (Fig. 2d). These features of CEUS suggested a diagnosis of renal malignancy. Subsequently a biopsy was performed under CEUS –guidance because of poor differentiation between the target and adjacent cysts in US. Then a further bolus of agent was injected, and a BARD automatic biopsy gun with an 18G percutaneous core needle biopsy was repeated under CEUS guidance to enhanced lesion (Fig. 3). Two tissue core samples with 2 cm in length were obtained from the targeted area (Fig. 4a). Histological examination of the specimen provided a diagnosis of diffuse large B-cell lymphoma (DLBCL) (Fig. 4b, c) with immunohistochemistry staining showing positive results for CD20, CD79, Bcl-2 and Bcl-6. Finally, clinical diagnosis was non-Hodgkin’s lymphoma (diffuse large cell, stage III B). Clinicians consider the prognosis very poor and communicate effectively with patients and their families. The patient consented to accepted chemotherapy consisting of rituximab and cyclophosphamide, hydroxydaunomycin, oncovin and prednisolone at the standard dose. Unfortunately, the patient developed deep vein thrombosis in the right side of his lower limb and pulmonary thromboembolism 2 weeks after one cycle chemotherapy. Despite active treatment, the patient’s condition has not been alleviated. Finally, the patient’s family gave up treatment and the patient was discharged.

## Discussion and conclusions

The incidence of PTLD in kidney transplant patients is approximately 1–10%, and it occurs most frequently during the first year after transplantation. Immunosuppression and EBV infection, are two major factors associated with the progression of PTLD [2] .The Gastrointestinal tract, allograft kidney and abdominal cavity are common sites of PTLD, but it is rare in the native kidney, which has been described in only three studies [10–12]. In previous reports, both US and CE-CT can clearly show the tumor and be used to characterize the tumor. The tumors showed persistent hypo-enhancement throughout the examination with CE-CT. The enhancement pattern of the tumor is the main difference from clear cell renal cell carcinoma, which present avid early hyper-enhancement and early wash-out throughout the examination [13, 14].

In our case, the tumor showed avid early hyper enhancement on CEUS; however, this was not visible on US and CE-CT. There are three possible explanations for this difference in our case: First, since the microvasculature of the atrophic renal parenchyma was reduced compared to a normal kidney, less iodine contrast agent entered the renal parenchyma [13]. In addition, the tumor was small in size, and there was much less iodine contrast agent entering the tumor. As a result, the tumor presented no enhancement on CE-CT, which caused a failure to distinguish tumors from surrounding cysts. Second, because CEUS contrast material is purely intravascular, it better correlates with the microvessel density of a tumor. Therefore, CEUS is even more sensitive for detection of hypovascular lesions than contrast-enhanced CT [15]. Although the native kidney perfusion reduced in our case, the microvascular density of the tumor is relatively more abundant than the peripheral renal parenchyma. As a result, the tumor appeared hyper enhanced compare to the surrounding renal parenchyma on CEUS. Third, the CE-CT scan often started at 35 s after the beginning of the injection, but the lesion showed peak enhancement at 33 s during CEUS examination. This may result in missing the peak enhancement phase of the tumor on CE-CT. In contrast, real-time CEUS can continually provide information about blood perfusion for the renal lesion after injection, which can offer more diagnostic clues to differential the tumor from adjacent cysts.

US-guided biopsy is a common clinical method to obtain tissue specimens for histopathological analysis [16]. However, this technique may be unsuccessful when the tumor is poorly differentiated from adjacent structures. CEUS is well placed to address this problem because of its capacity to differentiate between the altered vascularization of a tumor and surrounding structures. CEUS could help further confirm the tumor border and guide the needle to the target area. CEUS also can be used to differentiate enhanced active area from non-enhanced necrotic area. By directing the biopsy needle toward enhanced areas of the lesion, the sample from necrotic parts of the lesion can be reduced [17]. In a previous report, using the contrast agent, the lesion detection rate was increased from 77.3% with US to 92.0% with CEUS during the biopsy, with a 95.2% success rate for CEUS-guided biopsies of these lesions [18, 19]. According to these finding, we performed the biopsy in our patient under the guidance of CEUS. Finally, the pathology of the biopsy specimen confirmed the diagnosis of diffuse large B cell lymphoma.

In summary, CEUS can provide more useful information than CE-CT to detect and diagnose PTLDs derived from atrophic native kidneys; CEUS-guided biopsy can improve the diagnostic accuracy and success rate of percutaneous biopsy. We believe that CEUS and CEUS-guided biopsy may be an effective method for early screening and diagnosis of native kidney PTLD in kidney transplant patients.

## Data Availability

All data generated or analysed during this study are included in this published article.

## Abbreviations

CE-CT: Contrast-enhanced computed tomography
CE-MRI: Magnetic resonance imaging
CEUS: Contrast-enhanced ultrasonography
CMV: Cytomegalovirus
DLBCL: Diffuse large B-cell lymphoma
EBV: Epstein-Barr virus
FDG: Fluorodeoxyglucose F18
PET-CT: Positron emission tomography/computed tomography
PTLD: Post-transplant lymphoproliferative disorders
